# MiRNA-142-3p increases radiosensitivity in human umbilical cord blood mononuclear cells by inhibiting the expression of CD133

**DOI:** 10.1038/s41598-018-23968-1

**Published:** 2018-04-04

**Authors:** Fang Yuan, Lu Liu, Yonghong Lei, Yi Hu

**Affiliations:** 1 1Department of Oncology, Chinese PLA General Hospital, Beijing, 100853 China; 2 Department of Clinical Nutrition, Chinese PLA General Hospital, Beijing, 100853 China; 3 Department of Plastic Surgery, Chinese PLA General Hospital, Beijing, 100853 China

## Abstract

This study is to explore the molecular regulation mechanism of CD133 which is associated with malignancy and poor prognosis of blood system diseases. CD133+HUCB-MNC (human umbilical cord blood mononuclear cells) and CD133−HUCB-MNC were isolated and amplificated from umbilical cord blood, and then were exposed to different doses of radiation and subjected to a clonogenic assay. CCK-8 kit was used to detect cell viability, Annexin V-FITC/PI cell apoptosis detection kit was used for the detection of apoptotic cells and the BrdU assay was performed by flow cytometry. The expression of protein was analyzed by western blots. The profile of miRNA expression in response to radiation was examined and validated by RT-PCR. miR-142-3p inhibited the expression of CD133 in umbilical cord blood mononuclear cells to increase radiosensitivity. CD133+HUCB-MNC cells were more radioresistant compared with CD133−HUCB-MNC cells. CD133+HUCB-MNC cells showed higher p-AKT and p-ERK levels after radiation. And miR-142-3p acted on 3′UTR of CD133 mRNA to inhibit CD133 expression. Moreover, miRNA-142-3p mimic increased radiosensitivity in CD133+HUCB-MNC cells. Our results elucidated a novel regulation pathway in hematopoietic stem cells and suggested a potential therapeutic approach for blood system diseases therapy.

## Introduction

Radiotherapy is widely used for cancer treatment and the most common side effect is the bone marrow suppression^1^. Human umbilical cord blood mononuclear cells (hUCB-MNCs) is a suitable source of progenitor and stem cells, including subcomponents such as hematopoietic stem cells (HSCs), mesenchymal stem cell (MSCs), and endothelial progenitor cells (EPCs). Umbilical cord blood stem cells possess multi-differentiation potentials as mesoblast precursor^2^ which can differentiate into leukocytes, adipocytes, osteoblasts, muscle tendons and cardiocytes under the proper induction conditions^3^. Umbilical cord blood stem cells can differentiate into endothelial cells or MSCs both *in vitro* and *in vivo* and improve the poorly functioning organs^4^. Intracranial injection of hUCB-MNC during the hyperacute phase of ischemic stroke could improve cerebrovascular function and reduce infarct volume and behavioral deficits^5^.

The CD133 is a transmembrane glycoprotein which is considered as a significant cancer-associated cell surface marker. The expression of CD133 has been elevated in plenty of cancer cell types. CD133^+^ colon cancer cells showed chemoresistance to 5-fluorouracil by increasing the survivin expression^6^. CD133 facilitates the CSC-like properties by stabilizing EGFR-AKT signaling in Hepatocellular carcinoma cells (HCC)^7^. CD133 is a positive marker for a specific class of human cord blood-derived CD34-negative HSCs^8^. Radiotherapy leads to myelosuppression, while CD133 could resist radiotherapy-induced bone marrow suppression^9^. CD133+ cells were the source of most of the stem cells present in the HUCB-MNC, and CD133 was critical for the radiosensitivity of HUCB-MNCs^10^.

MicroRNAs (miRNAs) are key regulators for some cellular processes. Specific expression signatures have been found in different blood cell lineages and stages of HSC differentiation during hematopoiesis^11^. MiRNAs are small, non-coding RNAs found in the eukaryotes that control the expression of a large number of genes^12^ involved in commitment and differentiation of hematopoietic stem cells and tumorigenesis^13^. In particular, there has been a growing body of evidence supporting the role of miRNA in the regulation of CSCs recently^14^. For example, microRNA-139-5p regulates the proliferation of hematopoietic progenitors and is repressed during BCR-ABL-mediated leukemogenesis^15^.

Therefore, alterations in miRNAs can contribute to the inhibition of HSCs differentiation. MicroRNA-134-3p is a new potential inhibitor of human ovarian CSCs by targeting the RAB27A^16^. Wei-Wei Shen *et al*. reported that miR-142-3p functioned as a tumor suppressor by targeting CD133, ABCG2, and Lgr5 in colon cancer cells^17^. In this study, we identified miR-142-3p as a key modulator of CD133+ HUCB-MNC cells through pathways involving CD133, AKT and ERK pathways.

## Results

### *In vitro* isolation and amplification of CD133+HUCB-MNC and CD133−HUCB-MNC

**Figure 1:**
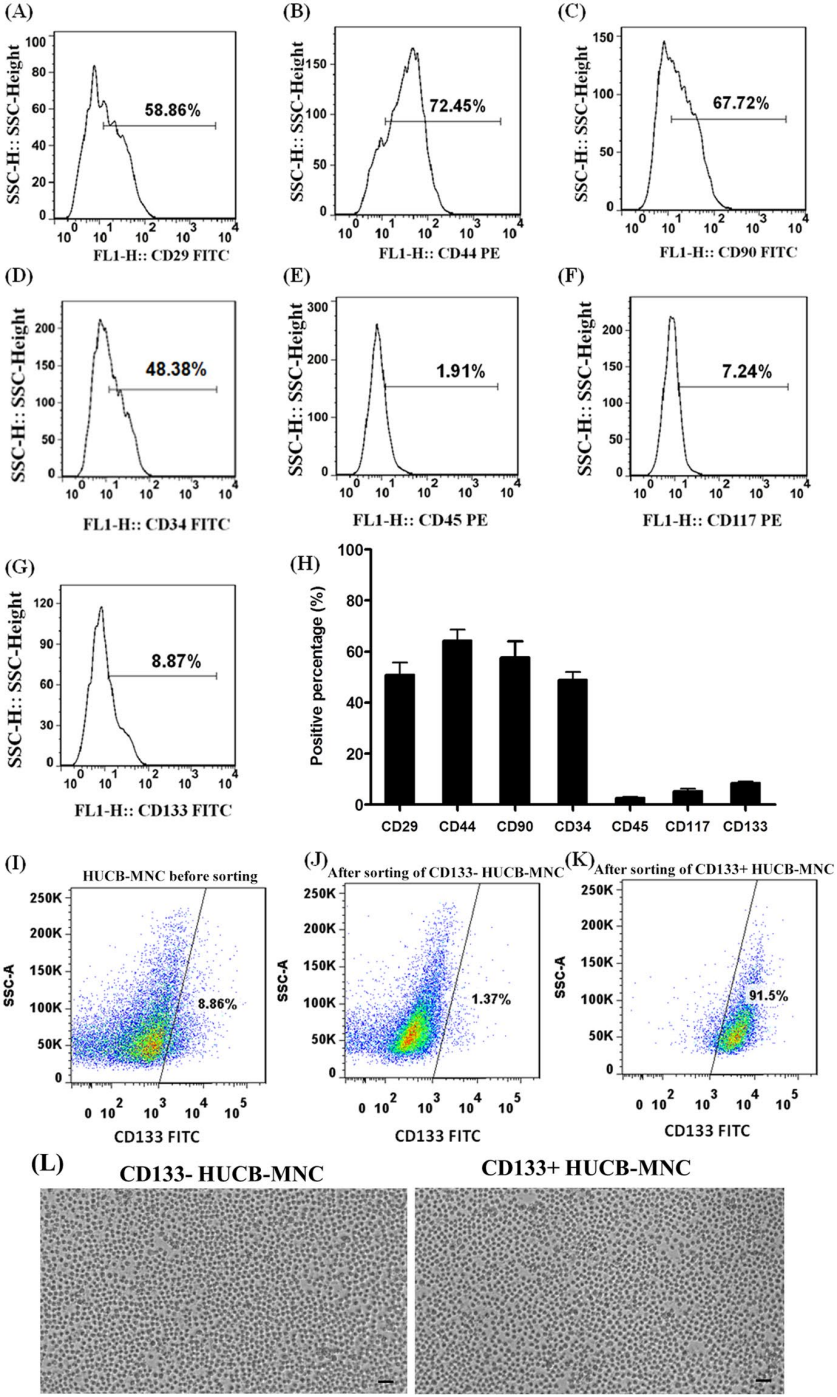
*In vitro* isolation and amplification of CD133+HUCB-MNC and CD133−HUCB-MNC. (**A**–**H**) Represent detection results of CD29, CD 44, CD34, CD90, CD45, CD117 and CD133 in HUCB-MNC cells. (**I**) represents cells before sorting. (**J**) Meant after sorting of CD133− cells. (**K**) represents cells after sorting of CD133+HUCB-MNC. (**L**) FACS-sorted CD133− cells (left) and CD133+ cells (right) were cultured in Iscove’s modified Dulbecco’s medium containing growth factors and cytokines. Scale bar = 50 μm. Assays were repeated three times. *P < 0.05, compared with the control group.

An appropriate source of HSCs is in the mononuclear cell (MNC) fraction of human umbilical cord blood (HUCB)^18^. HUCB-MNC cells were isolated from umbilical cord blood, and the surface maskers of these cells were analyzed by flow cytometry assay, including CD29 (51.02% ± 7.95%), CD44 (64.33% ± 7.45%), CD90 (57.63% ± 10.99%), CD34 (48.93% ± 5.32%), CD45 (2.67% ± 1.71%), CD117 (5.33% ± 1.69%) and CD133 (8.63% ± 0.67%) as shown in Fig. 1A–G and summarized in Fig. 1H. Furthermore, a stem cell enriched fraction (CD133+HUCB-MNC, 91.5% CD133-positive cells) and a stem cell depleted fraction (CD133−HUCB-MNC, 1.37% CD133-positive cells) of HUCB-MNC were sorted by flow cytometry (Fig. 1I–K). Cell culture images of FACS-sorted CD133+/− HUCB-MNC cells were shown in Fig. 1L.

### CD133+HUCB-MNC cells were more radioresistant compared with CD133−HUCB-MNC cells

**Figure 2:**
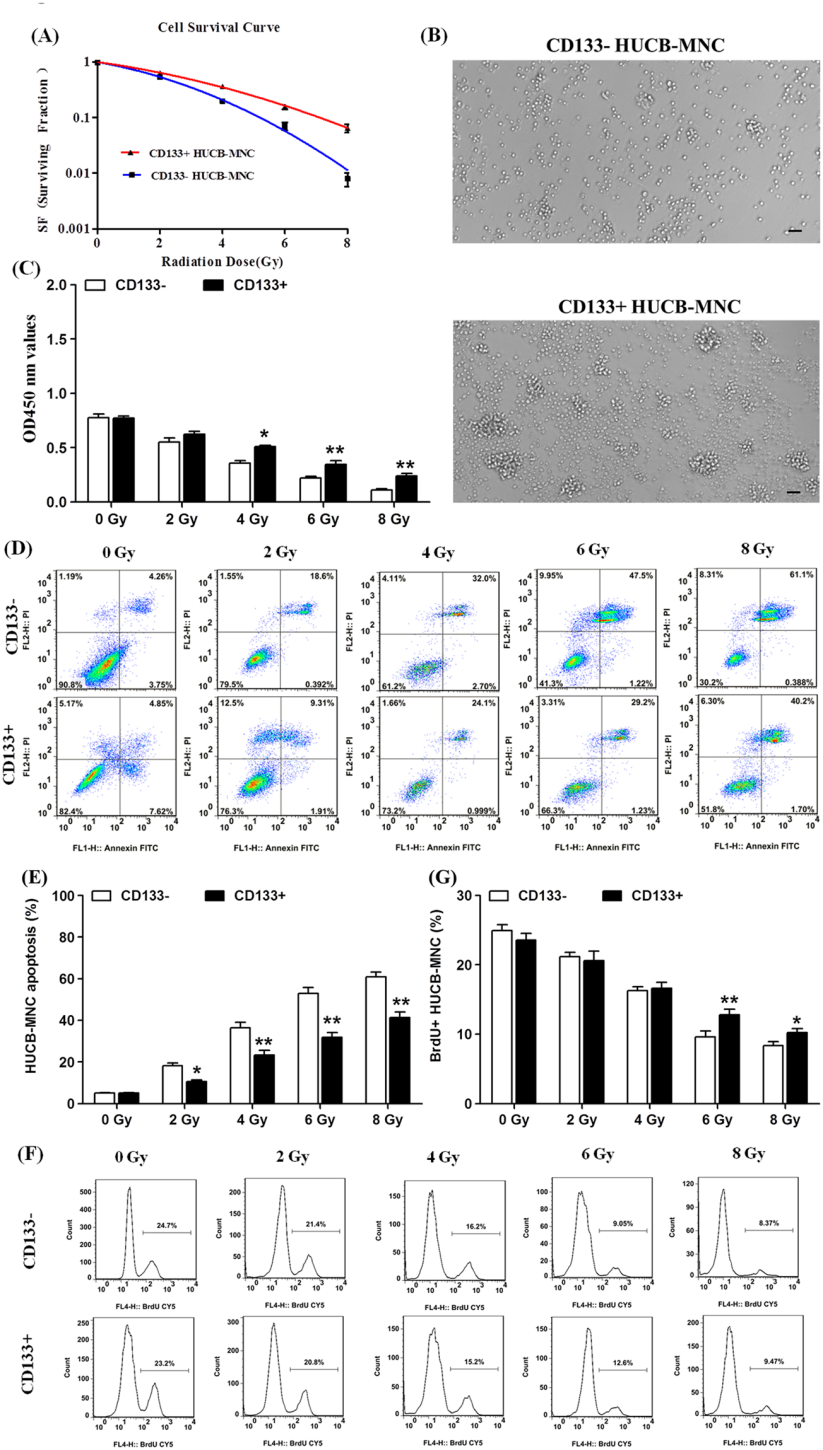
CD133+HUCB-MNC cells were more radioresistant compared with CD133−HUCB-MNC cells. (**A**) Represents the survival curves for each cell type. (**B**) Representative hematopoietic colony formation culture images of CD133− cells (upper) and CD133+ cells (lower) at 8 Gy radiation dose. Scale bar = 50 μm. (**C**) Represents the growth rate of CD133− cells and CD133+ cells at 4 Gy, 6 Gy and 8 Gy radiation dose. (**D**,**E**) Represent the apoptosis proportion in the two type’s cells. (**F**,**G**) Represent the the effect of CD133 on the cell cycle.

To explore the effect of CD133 on the cell response to radiation, CD133+HUCB-MNC cells and CD133−HUCB-MNC cells were exposed to different doses of radiation and subjected to a clonogenic assay. Cell colonies were counted and radiobiological parameters were calculated by survival curves for each cell type. Compared with the CD133−HUCB-MNC cells in the control group, the survival fractions of CD133+HUCB-MNC cells were much higher at 4 Gy (0.361 ± 0.057 vs 0.198 ± 0.034, p < 0.01), 6 Gy (0.158 ± 0.039 vs 0.071 ± 0.028, p < 0.001) and 8 Gy (0.065 ± 0.027 vs 0.008 ± 0.005, p < 0.001) radiation dose (Fig. 2A). Representative photomicrographs of cell colonies formed by CD133+/− HUCB-MNC cells at 8 Gy radiation dose were shown in Fig. 2B. The CCK8 proliferation assay results showed that the growth rate of CD133− cells was significantly decreased after radiation, compared with that of the CD133+ cells at 4 Gy (0.356 ± 0.040 vs 0.507 ± 0.023, p < 0.05), 6 Gy (0.221 ± 0.025 vs 0.347 ± 0.057, p < 0.01) and 8 Gy (0.110 ± 0.016 vs 0.238 ± 0.043, p < 0.01) radiation dose (Fig. 2C). We further looked into the effects of CD133 on the cell apoptosis after graded doses (0, 2, 4, 6, 8 Gy) radiation (Fig. 2D). As showed in Fig. 2D,E, the proportion of apoptosis decreased in the CD133+HUCB-MNC cells compared with CD133−HUCB-MNC cells at 2 Gy (10.53% ± 1.40% vs 18.12% ± 2.36%, p < 0.05), 4 Gy (22.03% ± 2.93% vs 36.36% ± 3.76%, p < 0.01), 6 Gy (31.96% ± 3.82% vs 52.87% ± 5.03%, p < 0.01) and 8 Gy (41.30% ± 3.58% vs 60.91% ± 3.98%, p < 0.01) radiation dose. Cell cycle is one of the crucial factors that affects radioresistance. To examine the CD133-mediated effect on the cell cycle, flow cytometry was performed to analyze the effect of CD133 on the cell cycle (Fig. 2F,G). The proportion of S phase cells increased in the CD133+HUCB-MNC cells compared with CD133−HUCB-MNC cells at 6 Gy (12.80% ± 1.41% vs 9.61% ± 1.49%, p < 0.05) and 8 Gy (10.23% ± 1.01% vs 8.39% ± 0.95%, p < 0.05) radiation doses. These results indicated that CD133+HUCB-MNC cells were more radioresistant compared with CD133−HUCB-MNC cells due to an increase in the proliferation rate, a decrease in the radiation induced apoptosis and prolonged S phase after radiation exposure.

### CD133+HUCB-MNC cells showed higher p-AKT and p-ERK levels after radiation

**Figure 3:**
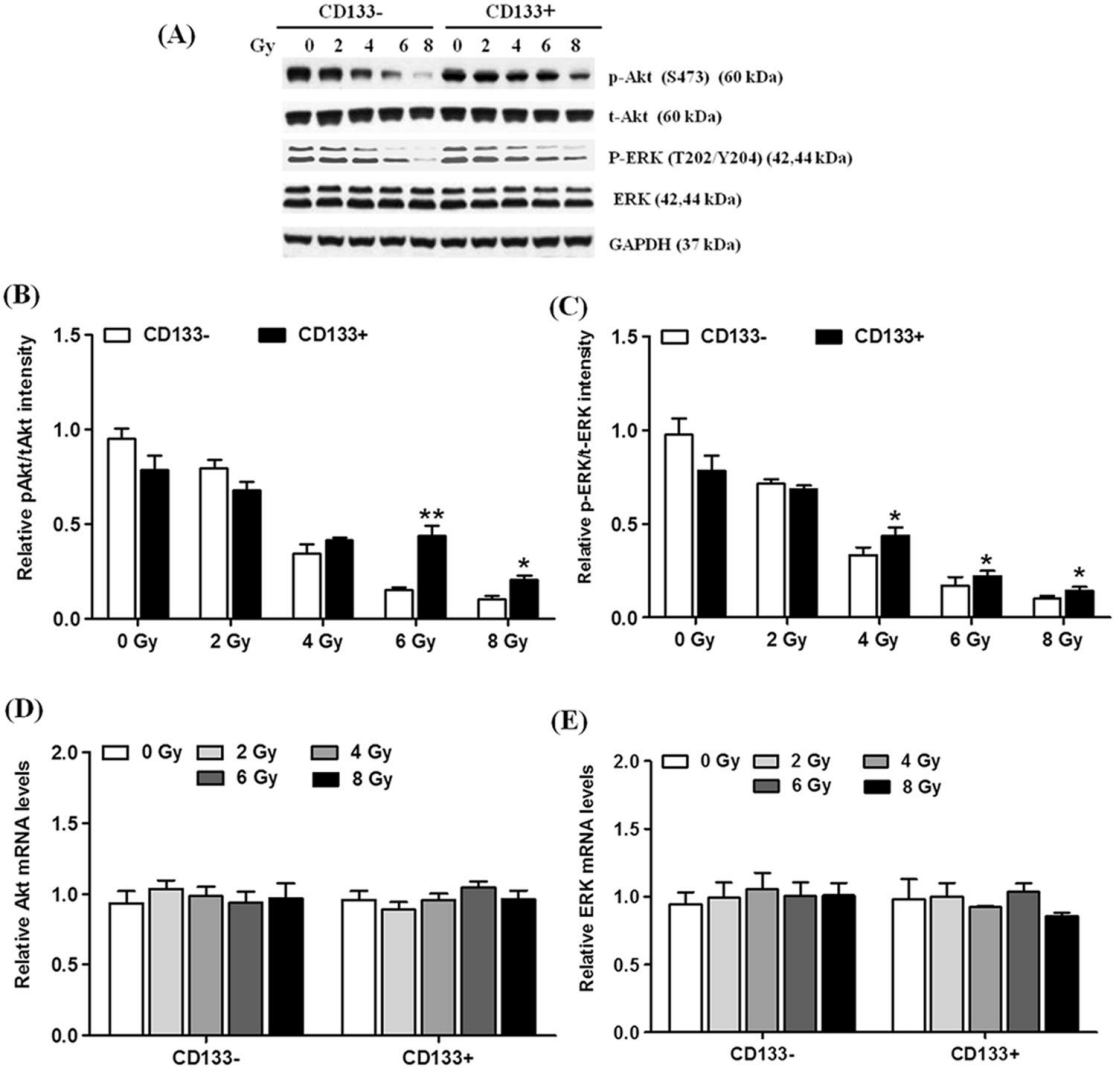
CD133+HUCB-MNC cells showed higher p-AKT and p-ERK levels after radiation. (**A**–**C**) Represent p-AKT, t-AKT, p-ERK (T202/Y204), t-ERK protein level. (**D**,**E**) Represent the mRNA level of AKT and ERK.

Signaling through the AKT and ERK pathways controls the cell proliferation. Western blots were used to analyze p-AKT, t-AKT, p-ERK (T202/Y204), t-ERK expression (Fig. 3A). The proportion of p-AKT/ t-AKT increased in the CD133+HUCB-MNC cells compared with CD133−HUCB-MNC cells at 6 Gy (0.442 ± 0.087 vs 0.150 ± 0.016, p < 0.01) and 8 Gy (0.207 ± 0.040 vs 0.103 ± 0.031, p < 0.05) radiation dose (Fig. 3B). The proportion of p-ERK (T202/Y204)/t−ERK increased in the CD133+HUCB-MNC cells compared with CD133−HUCB-MNC cells at 4 Gy (0.437 ± 0.076 vs 0.333 ± 0.071, p < 0.05), 6 Gy (0.220 ± 0.050 vs 0.170 ± 0.079, p < 0.05) and 8 Gy (0.143 ± 0.035 vs 0.100 ± 0.026, p < 0.05) radiation dose (Fig. 3C). However, no effect was observed on AKT and ERK mRNA levels (Fig. 3D,E). These results indicated that CD133+HUCB-MNC cells showed higher p-AKT and p-ERK levels after radiation.

### Screening of miRNAs Regulating CD133 Expression and miRNA validation by RT-PCR

**Figure 4:**
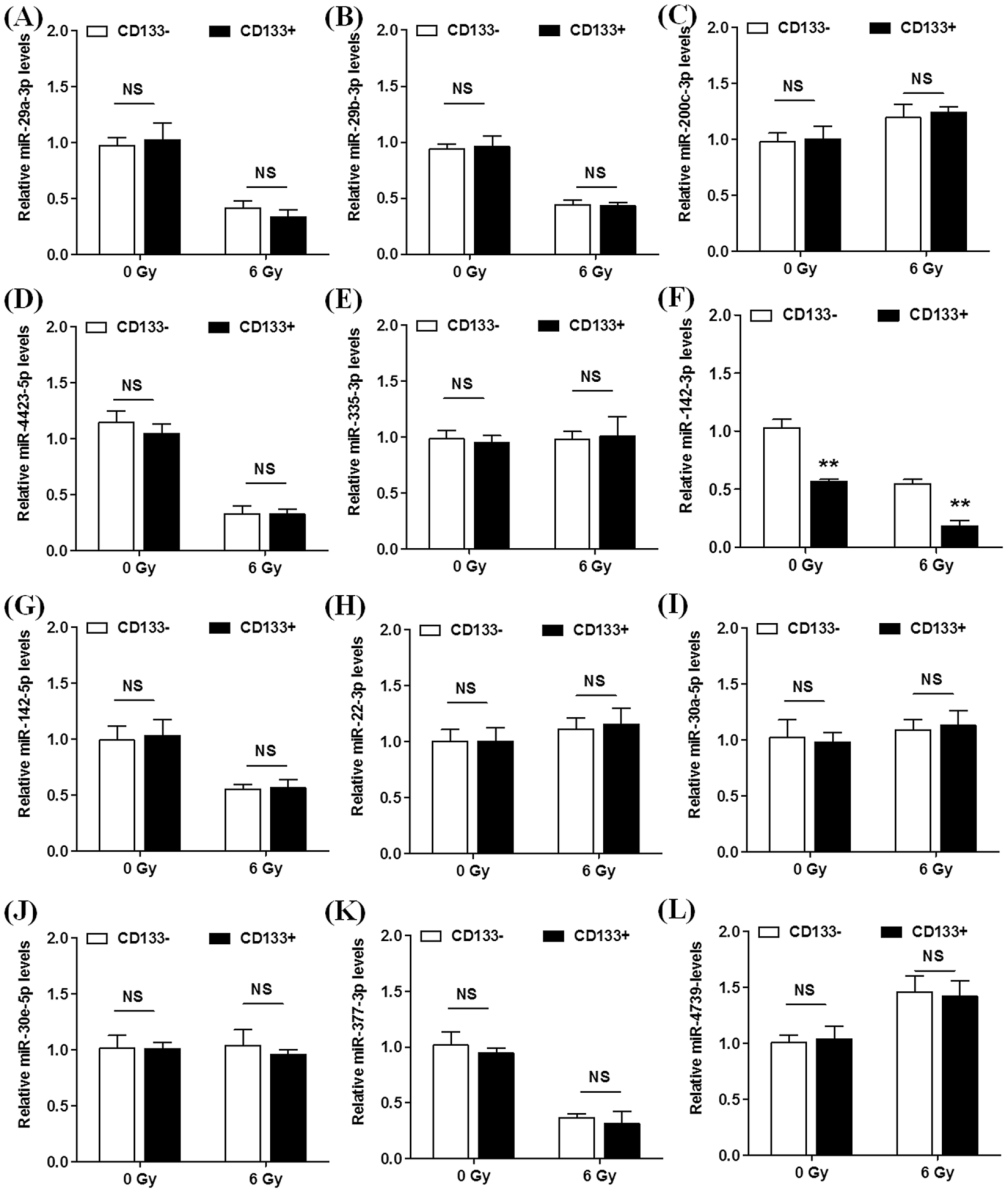
Screening of miRNAs Regulating CD133 Expression and miRNA validation by RT-PCR. (**A**–**L**) represent the expression level of hsa-miR-29a-3p, hsa-miR-29b-3p, hsa-miR-200c-3p, hsa-miR-4423-5p, hsa-miR-335-3p, hsa-miR-142-3p, hsa-miR-142-5p, hsa-miR-22-3p, hsa-miR-30a-5p, hsa-miR-30e-5p, hsa-miR-377-3p and hsa-miR-4739 between CD133+HUCB-MNC cells and CD133−HUCB-MNC cells at 0 and 6 Gy.

Combined with CD133 molecular regulation and human umbilical cord blood mononuclear cells expressed miRNA two aspects of information, the final confirmation of the following 12 miRNA^19^: hsa-miR-29a-3p (Fig. 4A), hsa-miR-29b-3p (Fig. 4B), hsa-miR-200c-3p (Fig. 4C), hsa-miR-4423-5p (Fig. 4D), hsa-miR-335-3p (Fig. 4E), hsa-miR-142-5p (Fig. 4G), hsa-miR-22-3p (Fig. 4H), hsa-miR-30a-5p (Fig. 4I), hsa-miR-30e-5p (Fig. 4J), hsa-miR-377-3p (Fig. 4K) and hsa-miR-4739 (Fig. 4L) showed no difference between CD133+HUCB-MNC cells and CD133−HUCB-MNC cells. MiR-142-3p levels were reduced in CD133 positive cells compared to cells that were CD133 negative (0.567 ± 0.032 vs 1.030 ± 0.125, p < 0.01). 6 Gy radiation decreased miR-142-3p expression both in CD133 positive and CD133 negative cells (0.180 ± 0.087 vs 0.547 ± 0.067, p < 0.01) (Fig. 4F).

### MiR-142-3p acted on 3′UTR of CD133 mRNA to inhibit CD133 expression

**Figure 5:**
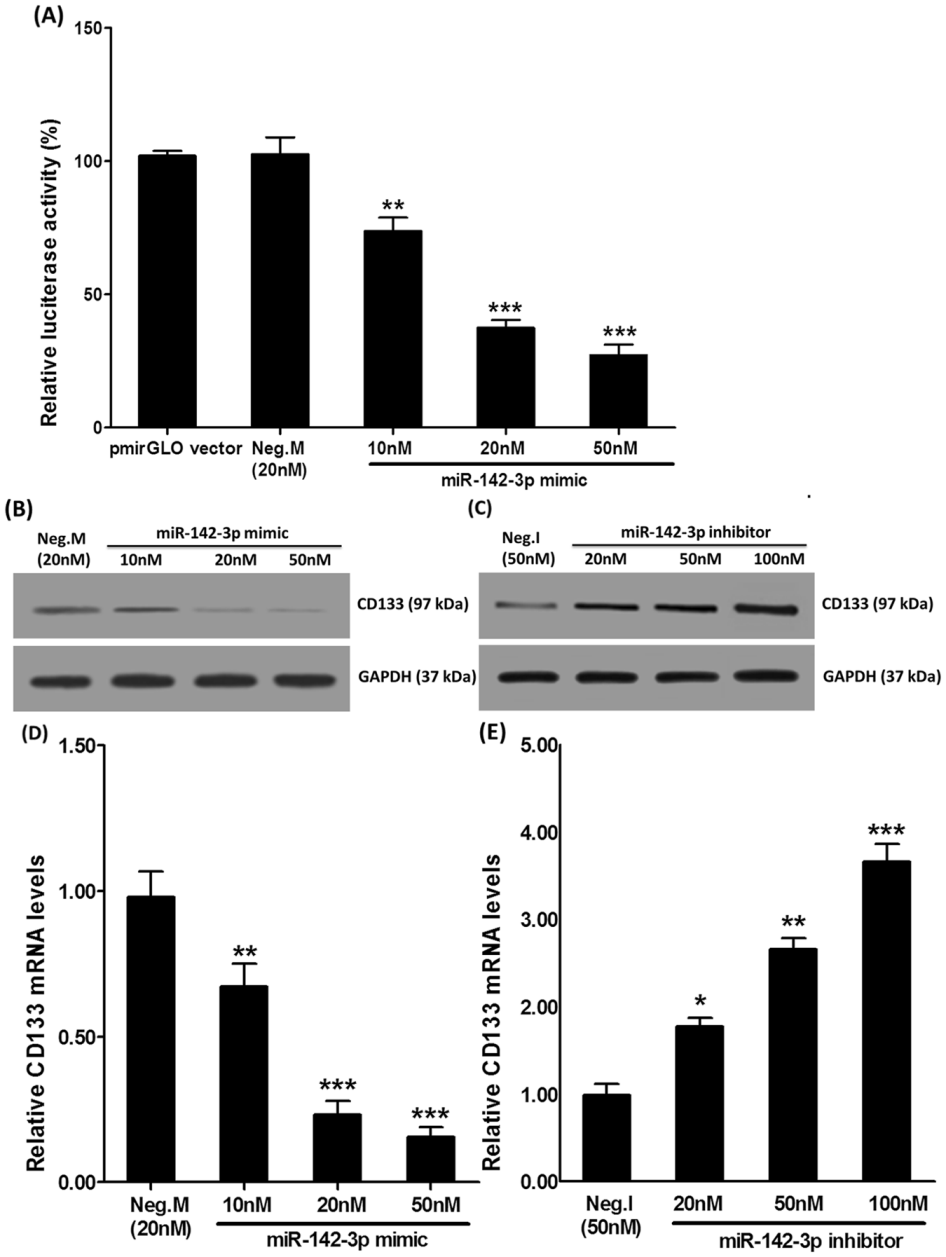
MiR-142-3p acted on 3′UTR of CD133 mRNA to inhibit CD133 expression. (**A**) MiR-142-3p directly targeted the 3′ untranslated region (3′-UTR) of CD133 mRNA. (**B**,**D**) Represent the Western Blot results CD133 expression in CD133+HUCB-MNC cells with miR-142-3p mimics. (**C**,**E**) Represent CD133 expression in CD133+HUCB-MNC cells with miR-142-3p inhibitors.

The results of a luciferase assay showed that miR-142-3p silenced CD133 by binding to the 3′-UTR of CD133 mRNA. MiR-142-3p mimic significantly reduced CD133 mRNA levels compared with the mimic control (10 nM, 73.787% ± 8.724% vs 102.567% ± 11.073%, p < 0.01; 20 nM, 37.467% ± 5.054% vs 102.567% ± 11.073%, p < 0.001; 50 nM, 27.200% ± 6.873% vs 102.567% ± 11.073%, p < 0.001), and there was no difference between the mimic control group and the pmirGLO vector group (102.567% ± 11.073% vs 102.033% ± 3.099%, p > 0.05) (Fig. 5A). Transfection of miR-142-3p mimics in CD133+HUCB- MNC cells downregulated CD133 expression compared with the mimic control both in mRNA (10 nM, 0.672 ± 0.136 vs 0.979 ± 0.152, p < 0.01; 20 nM, 0.233 ± 0.082 vs 0.979 ± 0.152, p < 0.001; 50 nM, 0.155 ± 0.059 vs 0.979 ± 0.152, p < 0.001) and protein levels (Fig. 5B,D). And transfection of miR-142-3p inhibitors in CD133+HUCB-MNC cells increased CD133 expression (20 nM, 1.779 ± 0.164 vs 0.992 ± 0.216, p < 0.05; 50 nM, 2.662 ± 0.213 vs 0.992 ± 0.216, p < 0.01; 100 nM, 3.661 ± 0.351 vs 0.992 ± 0.216, p < 0.001) (Fig. 5C,E). Therefore, miR-142-3p acted on 3’UTR of CD133 mRNA to inhibit CD133 expression.

### miRNA-142-3p mimic increased radiosensitivity in CD133+HUCB-MNC cells

**Figure 6:**
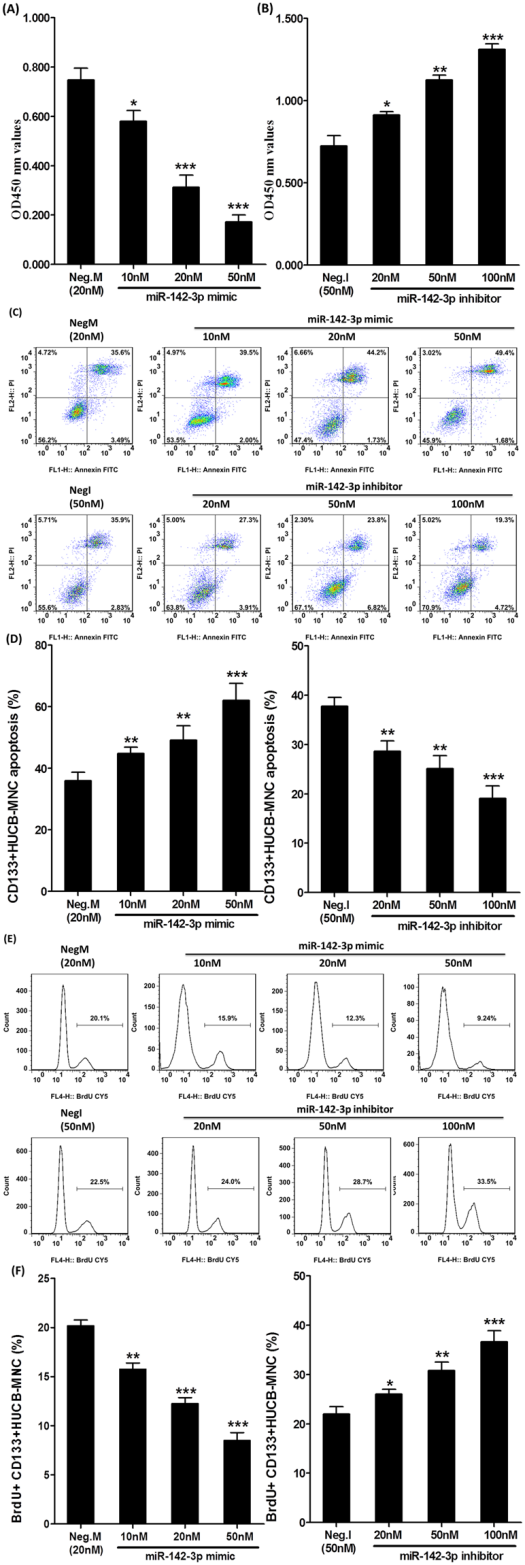
miRNA-142-3p mimic increased radiosensitivity in CD133+HUCB-MNC cells. (**A**,**B**) Represents the CD133+HUCB-MNC cells’ proliferation rate in CD133+HUCB-MNC cells transfected with indicated dose of hsa-miR-142-3p mimics or inhibitors. (**C**,**D**) Represent apoptosis proportion in CD133+HUCB-MNC cells transfected with indicated dose of hsa-miR-142-3p mimics or inhibitors. (**E** and **F**) Represent the proportion of S phase cells at 6 Gy radiation dose in CD133+HUCB-MNC cells transfected with indicated dose of hsa-miR-142-3p mimics or inhibitors.

**Figure 7:**
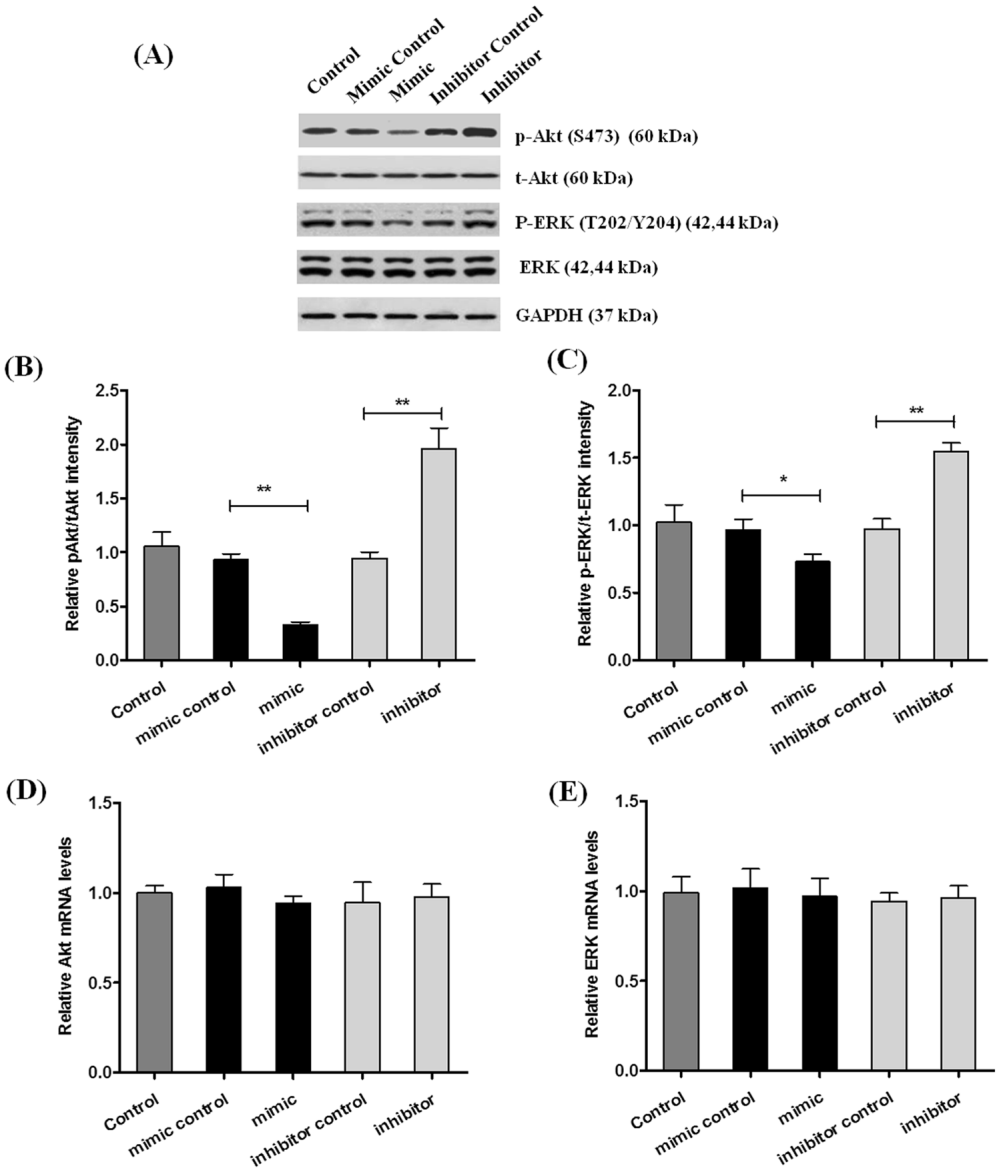
miRNA-142-3p mimic inhibited the proportion of p-ERK (T202/Y204)/t-ERK. (**A**–**C**) Represent the proportion of p-AKT/t-AKT and p-ERK (T202/Y204)/t-ERK at 6 Gy radiation dose in control CD133+HUCB-MNC cells and CD133+HUCB-MNC cells transfected with 20 nM hsa-miR-142-3p mimic, 20 nM mimic control, 50 nM hsa-miR-142-3p inhibitor, or 50 nM inhibitor control. (**D**,**E**) Represent AKT and ERK mRNA level.

To explore the effect of miRNA-142-3p on radiosensitivity in CD133+HUCB-MNC cells, both miRNA-142-3p mimic and miRNA-142-3p inhibitor were used to detect the effects on cell proliferation, cell apoptosis, cell cycle, AKT and ERK protein and mRNA levels after the 6 Gy radiation. MiRNA-142-3p mimic reduced CD133+HUCB-MNC cells’ proliferation rate compared with the mimic control group (10 nM, 0.580 ± 0.077 vs 0.747 ± 0.083, p < 0.05; 20 nM, 0.313 ± 0.085 vs 0.747 ± 0.083, p < 0.001; 50 nM, 0.172 ± 0.049 vs 0.747 ± 0.083, p < 0.001) (Fig. 6A), while miRNA-142-3p inhibitor increased the proliferation rate compared with the inhibitor control group (20 nM, 0.912 ± 0.038 vs 0.725 ± 0.107, p < 0.05; 50 nM, 1.126 ± 0.049 vs 0.725 ± 0.107, p < 0.01; 100 nM, 1.311 ± 0.059 vs 0.725 ± 0.107, p < 0.001) (Fig. 6B). We further explored the effect of miRNA-142-3p on cell apoptosis after 6 Gy radiation exposures. As showed in Fig. 6C,D, the proportion of apoptosis increased in the miRNA-142-3p mimic group compared with mimic control group (10 nM, 44.819% ± 3.572% vs 35.897% ± 4.880%, p < 0.01; 20 nM, 49.160% ± 8.047% vs 35.897% ± 4.880%, p < 0.01; 50 nM, 62.032% ± 9.634% vs 35.897% ± 4.880%, p < 0.001), while miRNA-142-3p inhibitor reduced the proportion of apoptosis compared with inhibitor control group (20 nM, 28.629% ± 3.572% vs 37.745% ± 3.094%, p < 0.01; 50 nM, 25.117% ± 4.496% vs 37.745% ± 3.094%, p < 0.01; 100 nM, 19.091% ± 4.371% vs 37.745% ± 3.094%, p < 0.001). The proportion of S phase cells decreased in the miRNA-142-3p mimic group compared with mimic control group at 6 Gy radiation dose (10 nM, 15.759% ± 1.096% vs 20.187% ± 1.002%, p < 0.01; 20 nM, 12.273% ± 1.030% vs 20.187% ± 1.002%, p < 0.001; 50 nM, 8.502% ± 1.382% vs 20.187% ± 1.002%, p < 0.001), and miRNA-142-3p inhibitor upregulated the proportion of S phase cells compared with inhibitor control group (20 nM, 25.967% ± 1.812% vs 21.987% ± 2.539%, p < 0.05; 50 nM, 30.757% ± 3.025% vs 21.987% ± 2.539%, p < 0.01; 100 nM, 36.595% ± 3.974% vs 21.987% ± 2.539%, p < 0.001) (Fig. 6E,F). The proportion of p-AKT/t-AKT decreased in the miRNA-142-3p mimic group compared with mimic control group at 6 Gy radiation dose (20 nM, 0.330 ± 0.046 vs 0.930 ± 0.095, p < 0.01) (Fig. 7A,B). What’s more, the proportion of p-ERK (T202/Y204)/t- ERK decreased in the miRNA-142-3p mimic group compared with mimic control group at 6 Gy radiation dose (20 nM, 0.730 ± 0.095 vs 0.967 ± 0.134, p < 0.05) (Fig. 7A,C). And the miRNA-142-3p inhibitor showed opposite effects on the p-AKT and p-ERK (T202/Y204) levels. However, no effect was observed on AKT and ERK mRNA levels (Fig. 7D,E). The results above indicated that miRNA-142-3p mimic increased radiosensitivity in CD133+HUCB-MNC cells.

## Discussion

Umbilical cord blood is considered as an alternative source for stem cell transplantation and therapy due to its hematopoietic and mesenchymal properties^20^. For both HSCs and leukemia stem cells (LSCs), specific gene expression programs distinguish them from their differentiated progenies^21^. Human HSCs are a primary target of radiation-induced leukemogenesis and provide a correlation cellular model for evaluating the risk of cancer.

The CD133 is considered as a tumor marker in many cancer types. The therapeutic capacity of HUCB-MNC and stem cells is documented in animal models of focal cerebral ischemia, CD133+HUCB-MNC reduced neuronal apoptosis in the range of normoxic controls^18^. Radiotherapy leads to myelosuppression, but CD133 could resist radiotherapy-induced bone marrow suppression. CD133 can be phosphorylated on cytoplasmic tyrosine-828 by the Src family kinases^22^. And the Src family kinases can be activated rapidly by multiple cell surface receptors combination and regulate many cellular processes that include proliferation, differentiation and adhesion^23^. In our study, the results indicated that CD133+HUCB-MNC cells were more radioresistant compared with CD133−HUCB-MNC cells due to an increase in the proliferation rate, a decrease in the radiation induced apoptosis and prolonged S phase after radiation exposure, and CD133+HUCB-MNC cells showed higher p-AKT and p-ERK levels after radiation. CD133+ is a marker of human progenitor and hematopoietic stem cells; repair signaling in CD133+UCBC was different from CD133− UCBC and PBL. These differences included higher 53BP1 recruitment and lower endogenous DSB levels^24^. CD133-p85 interaction promotes tumorigenic capacity of glioma stem cells by activating of PI3K/Akt pathway^25^. CD133+cells are used for treatment of leukemia, liver regeneration, neurodegenerative diseases and myocardial infarction^26^.

MicroRNAs are post-transcriptional regulators which bind to complementary sequences on target messenger RNA, usually leading to translational repression or target degradation and gene silencing^27^. MiRNAs were proved to play important roles in control of multifaceted of HSC and LSC biology^28^. Recent studies have showed that roles of miRNAs in reaction to physiologic and pathologic stress such as DNA damage in fully developed tissues. For example, micro-RNA30c negatively regulates REDD1 expression in human hematopoietic and osteoblast cells after irradiation^1^. Our results of a luciferase assay showed that miR-142-3p silenced CD133 by binding to the 3′-UTR of CD133 mRNA, and miRNA-142-3p mimic increased radiosensitivity in CD133+ HUCB-MNC cells. Therapeutic delivery of miR142-3p in ATRT cells suggested effective reduction in its lethality by repressing tumor growth, inhibiting invasion, enhancing radiosensitivity, and prolonging survival time in orthotropic-transplanted immunocompromised mice^29^. Moreover miR-142-3p directly targets CD133 to regulate its ability to confer cancer and stem cell-like features in HCC^21^.

## Conclusions

In conclusion, CD133+HUCB-MNC cells were more radioresistant compared with CD133−HUCB-MNC cells.CD133+HUCB-MNC cells showed higher p-AKT and p-ERK levels after radiation. The results of a luciferase assay showed that miR-142-3p silenced CD133 by binding to the 3′-UTR of CD133 mRNA. Overexpression of miR-142-3p in human CD133+HUCB-MNC cells can not only inhibit the expression of CD133 but also can inhibit the proliferation *in vitro*, arrest cell cycle progression, and promote apoptosis. This study gains an insight into the mechanism of radiotherapy resistance and gives a new strategy for blood system disease therapies in the future. Identification of the markers under the control of miRNA-142-3p and their regulation in CD133+HUCB-MNC cells may help develop efficient targeted treatments, improved diagnostic methods, and better prognostic evaluation.

## Methods

### Isolation of primary umbilical cord blood mononuclear cells

Blood samples (about 50 mL) were collected from three fresh placentas with attached umbilical cords by gravity flow. MNCs were isolated by density gradient centrifugation over Biocoll (Biochrom, Berlin, Germany) for 30 min at 400 × g and washed three times in phosphate buffered saline (PBS) (Biochrom)^2^. MNCs were counted and incubated for 30 min at 4 °C with anti-human CD133 (eBioscience, CA, USA). CD133+/− MNCs were purified by FACS (fluorescence-activated cell sorter) sorting using a FACSAria III flow cytometer (BD Biosciences, CA, USA). The FACS-sorting purity were more than 90%. The mononuclear cells were inoculated in Iscove’s modified Dulbecco’s medium supplied with 10% FBS, 15 ng/mL IL-3, and 5 ng/mL Granulocyte-macrophage colony-stimulating factor (GM-CSF), and cultured in a humidified 5% CO_2_ atmosphere at 37 °C. The medium was replaced by half at a 3-day interval until the attached cells grew to 80% confluence, and then used for the following experiments^30^. All experimental procedures were approved by Laboratory Animal Ethics Committee of Chinese PLA general hospital. The principles outlined in the ARRIVE (Animal Research: Reporting of *in vivo* Experiments) guideline and Basel declaration (including the 3 R concept) were considered when planning experiments. Written informed consent was obtained from all subjects.

### Flow Cytometry

The cells were examined for immune phenotype (CD29, CD44, CD90, CD34, CD45, CD133, and CD117). Cells were collected for cell-surface staining. The following reagents were used for surface staining: Anti-Integrin beta 1 (Alternative name: CD29) antibody [MEM-101A] (FITC) (ab21845) Anti-CD44 antibody [F10-44-2] (Phycoerythrin) (ab82529), Anti-CD90/Thy1 antibody [F15-42-1] (FITC) (ab11155), Anti-CD45 antibody [MEM-28], prediluted (Phycoerythrin) (ab134202), eBioscience 11-1339-42 FITC human CD133, Anti-CD34 antibody [4H11[APG]] (FITC) (ab18227), Anti-c-Kit (Alternative name: CD117) antibody [104D2] (Phycoerythrin) (ab111245). Flow cytometry data for each of the experiments were acquired using BD FACSCalibur (BD Immunocytometry Systems). Data analysis was performed with FlowJo software (Tree Star)^31^.

### Hematopoietic colony formation assay

A volume of 1 × 10^5^/ml CD133+HUCB-MNC Cells or 1 × 10^5^/ml CD133−HUCB-MNC Cells were plated in 12-well plate culture flasks coated with methylcellulose and 10 ng/ml Granulocyte colony stimulating factor (G-CSF). After 24 h, the cells were irradiated with graded doses (0, 2, 4, 6, 8 Gy) using X-ray generator (Primus High-Energy Siemens) at a dose rate of 2 Gy/min. After 10 days of incubation in a humidified 5% CO_2_ atmosphere at 37 °C, the colonies were fixed. Those colonies containing ≥50 cells were scored as viable colonies. The data were fit into the single-hit multi-target model, and the survival curve of each group was demonstrated by Graphpad prism 6.0 software (San Diego, CA, USA)^32^. The hematopoietic colony formation assays were performed in triplicate.

### CCK8 Cell proliferation assay

CCK-8 kit (Beyotime, China) was used to detect cell viability^6^. Briefly, CD133+HUCB-MNC cells or CD133−HUCB-MNC cells were seeded into 96-well plate (Corning Costar, Cambridge, MA) at a density of 0.2 × 10^4^ cells per well. After 24 h, the cells were irradiated with graded doses (0, 2, 4, 6, 8 Gy) using X-ray generator (Primus High-Energy Siemens) at a dose rate of 2 Gy/min. 14 h after radiation, 10 μL of CCK-8 solution was added, followed by incubation for 3 h at 37 °C. The optical density (OD) was measured at 450 nm to reflect the cell viability. Detection was done in 6 wells per group, and blank controls were also detected. The proliferation assays were performed in triplicate.

### Detection of cell apoptosis by flow cytometry

Annexin V-FITC/PI cell apoptosis detection kit (BD Biosciences, SanJose, CA) was used for the detection of apoptotic cells. Cells in logarithmic growth phase were used to prepare single cell suspension at 1 × 10^6^ cells/ml. After addition of propidium iodide (PI) and FITC Annexin V, the apoptotic cells were detected by flow cytometry (BD Biosciences), and apoptosis rate was calculated. Experiment was done thrice, and data were analyzed with Flowjo (tree star)^33^.

### BrdU labeled cell cycle analysis

Briefly, cells were incubated with 10 μM BrdU for 2 h and then were fixed with 4% paraformaldehyde. After rinsed with PBS, cells were treated with 2 N HCl for 30 min. Nonspecific binding was blocked in 5% bovine serum albumin (BSA) for 1 h at room temperature. Cells were respectively incubated with mouse anti-BrdU (1:200, Millipore, MAB3222) overnight at 4 °C one after another. Then followed by three times washes in PBS, they were incubated with Goat Anti-Mouse IgG H&L (Cy5®) preadsorbed (ab6563) for 1 h at room temperature. Immunofluorescence was observed using flow cytometry.

### Western blot

Total proteins were extracted after treatment using a standard method and the protein concentrations were determined using Bradford method. 30 μg proteins were electrophoresed on a 12% SDS-polyacrylamide gels, followed by transferring to a nitrocellulose membrane. Membrane was incubated with the following primary antibodies: Phospho-Akt (Ser473) (D9E) XP® Rabbit mAb (1:1000, Cst 4060), Anti-T-Akt (1:1000, Cst 9272), Anti-ERK 1/2 (1:1000, cst #9102), Anti-P-ERK 1/2 (1:2000, cst4370) or Anti-GAPDH(1:1000, cst 5174), Anti-CD133 antibody - Stem Cell Marker (ab16518, 1:500). Blots were washed with TBST, then followed by the addition of the secondary antibodies goat anti-rabbit IgG-HRP conjugate (1:2000, ab6721) at 37 °C for 1 h. Bound antibodies were detected with enhanced chemiluminescence (ECL) reagents (Amersham, Cleveland, OH, USA) and membranes were exposed to Hyperfilm (Amersham, Cleveland, OH, USA). The intensity values of the protein were measured by ImageJ (National Institutes of Health) and normalized to that of GAPDH and expressed as a relative ratio.

### RNA isolation and quantitative real-time polymerase chain reaction

After treatment, total RNA was prepared by using total RNA Kit (R6934, Omega Bio-tek Inc., GA, U.S.A.) and cDNA was synthesized from 5 μg RNA in cDNA Synthesis Kit (K1622, Fermentas International Inc., Canada) according to the manufacturer’s instructions Each PCR was performed in triplicate in a final volume of 20 μL solution: 10 μL of SYBR Green dye, 1 μL of diluted cDNA products, 0.2 μM of each paired primer, 8.6 μL deionized water. Protocols were as follows: initial denaturation for 5 min at 94 °C, followed by 40 cycles denaturation for 30 s at 94 °C and extension for 30 s at 58 °C. The last cycle for dissociation of SYBR Green probe was 15 s at 95 °C, 30 s at 60 °C and 15 s at 95 °C. The primer sequences for Akt were: Forward: 5′-AGCGACGTGGCTATTGTGAAG-3′, Reverse: 5′-GCCATCATTCTTGAGGAGGAAGT-3′. The primer sequences for Erk were: Forward: 5′-TCACACAGGGTTCCTGACAGA-3′, Reverse: 5′-ATGCAGCCTACAGACCAAATATC-3′. The primer sequences for the control GAPDH were: Forward: 5′-CTGGGCTACACTGAGCACC-3′, Reverse: 5′-AAGTGGTCGTTGAGGGCAATG-3′. The microRNA primer sequences were shown in Supplementary table 1. Assays were performed in triplicate with the ABI7500 instrument. All data were normalized by GAPDH. Gene expression data was analyzed by the 2^−△△CT^ method.

### Screening of miRNAs Regulating CD133 Expression

An approach combining bioinformatic prediction of miRNA targets with mRNA expression profiling was used to search for putative biologically enriched functions and networks. MiRNA prediction tools included: miRanda^34^, RNA22^35^, TargetScan^36^, and MiRWalk^37^ Combined with CD133 molecular regulation and human umbilical cord blood mononuclear cell s expressed miRNA two aspects of information, the final confirmation of the following 12 miRNA^19^: hsa-miR-29a-3p, hsa-miR-29b-3p, hsa-miR-200c-3p, hsa-miR-4423-5p, hsa-miR-335-3p, hsa-miR-142-3p, hsa-miR-142-5p, hsa-miR-22-3p, hsa-miR-30a-5p, hsa-miR-30e-5p, hsa-miR-377-3p and hsa-miR-4739 (Supplementary Table 2).

### miRNA validation by RT-PCR

Total RNA was reverse transcribed with miScript II RT kit (Qiagen) according to manifacturer’s instructions. qPCR was performed in triplicate on Rotor-GeneQ (Qiagen) using miScript SYBR Green PCR kit (Qiagen). Data were normalized to RNU6 microRNA expression using the Ct method. The microRNA primer sequences are illustrated in Supplementary Table 1^11^.

### Dual luciferase reporter gene assay

The 3′UTR of CD133 (sense: 5′-GCTAGCggaggcggaa ttcttgaccg-3′, antisense: 5′-GTCGACt caggcatgcc accccgacca cccgaggctg tg-3′) was amplified with PCR. Genomic DNA was extracted from the blood, and the 3′UTR of CD133 containing miR-142-3p binding site was amplified. The PCR products were inserted into pmirGLO vector to acquire recombinant CD133-wt. CD133+HUCB-MNC cells were seeded into 12-well plates, and the vector (CD133-wt) was trasnfected into CD133+HUCB-MNC cells with hsa-miR-142-3p mimic (micrON™ hsa-miR-142-3p mimic, Standard, 5nmol, miR10000434-1-5) or negative control mimic (neg. M) (micrON™ mimic Negative Control #24, Standard, 5nmol, miR01201-1-5) by using LipofectamineTM 2000 kit (Invitrogen, Carlsbad, CA, USA). 24 h later, dual luciferase reporter gene assay kit (Promega) was used to detect the dual luciferase signals. The reagents for hsa-miR-142-3p inhibition were: hsa-miR-142-3p inhibitor (micrOFF™ hsa-miR-142-3p inhibitor, Standard, 5nmol, miR20000434-1-5), and negative control inhibitor (neg. I) (micrOFF™ inhibitor Negative Control #24, Standard, 5nmol, miR02201-1-5).

### Statistical analysis

Statistical analysis was performed with SPSS17.0 statistical software (SPSS Inc., Chicago, IL, USA). Data were expressed as mean ± SEM. One-way ANOVA in conjunction with least significant difference test was performed to determine statistical significance (*P* < 0.05). The difference was considered significantly if p < 0.05. *means p < 0.05, **means p < 0.01 and ***means p < 0.001.

### Data Availability

All data generated or analyzed during this study are included in this published article.

## Electronic supplementary material

Supplementary table
