# Supplementary material for: MiRNA-142-3p increases radiosensitivity in human umbilical cord blood mononuclear cells by inhibiting the expression of CD133
Source: Sci Rep. 2018 Apr 4;8:5674. doi: 10.1038/s41598-018-23968-1 (PMC5884857; doi:10.1038/s41598-018-23968-1)
Supplement: Supplementary file 1 — Supplementary table [file 41598_2018_23968_MOESM1_ESM.docx]

**MiRNA-142-3p increases radiosensitivity in human umbilical cord blood mononuclear cells by inhibiting the expression of CD133**

Fang Yuan1#, Lu Liu2#, Yonghong Lei3*, Yi Hu1*

1Department of Oncology, Chinese PLA General Hospital, Beijing, 100853; 2Department of Clinical Nutrition, Chinese PLA General Hospital, Beijing, 100853; 3Department of Plastic Surgery, Chinese PLA General Hospital, Beijing, 100853

# These authors contributed equally to this work.

***** **Correspondence and requests for materials should be addressed to:** L.Y. (lyhhlx@126.com) or H.Y. (huyi0401@aliyun.com)

**Table 1. microRNA primer sequences.**

| microRNA | primer sequences | Product number |
| --- | --- | --- |
| hsa-miR-142-5p | [Bulge-Loop™hsa-miR-142-5pqRT-PCR Primer Set, Standard, 100rxns](http://www.ribobio.com/sitecn/product_info.aspx?id=201064) | [miRQ0000433-1-1](http://www.ribobio.com/sitecn/product_info.aspx?id=201064) |
|  | Mature miRNA sequences: CAUAAAGUAGAAAGCACUACU |  |
| hsa-miR-22-3p | [Bulge-Loop™hsa-miR-22-3pqRT-PCR Primer Set, Standard, 100rxns](http://www.ribobio.com/sitecn/product_info.aspx?id=200688) | [miRQ0000077-1-1](http://www.ribobio.com/sitecn/product_info.aspx?id=200688) |
|  | Mature miRNA sequences: AAGCUGCCAGUUGAAGAACUGU |  |
| hsa-miR-30a-5p | [Bulge-Loop™hsa-miR-30a-5pqRT-PCR Primer Set, Standard, 100rxns](http://www.ribobio.com/sitecn/product_info.aspx?id=200708) | [miRQ0000087-1-1](http://www.ribobio.com/sitecn/product_info.aspx?id=200708) |
|  | Mature miRNA sequences: UGUAAACAUCCUCGACUGGAAG |  |
| hsa-miR-30e-5p | [Bulge-Loop™hsa-miR-30e-5pqRT-PCR Primer Set, Standard, 100rxns](http://www.ribobio.com/sitecn/product_info.aspx?id=201420) | [miRQ0000692-1-1](http://www.ribobio.com/sitecn/product_info.aspx?id=201420) |
|  | Mature miRNA sequences: UGUAAACAUCCUUGACUGGAAG |  |
| hsa-miR-377-3p | [Bulge-Loop™hsa-miR-377-3pqRT-PCR Primer Set, Standard, 100rxns](http://www.ribobio.com/sitecn/product_info.aspx?id=201472) | [miRQ0000730-1-1](http://www.ribobio.com/sitecn/product_info.aspx?id=201472) |
|  | Mature miRNA sequences: AUCACACAAAGGCAACUUUUGU |  |
| hsa-miR-4739 | [Bulge-Loop™hsa-miR-4739qRT-PCR Primer Set, Standard, 100rxns](http://www.ribobio.com/sitecn/product_info.aspx?id=207350) | [miRQ0019868-1-1](http://www.ribobio.com/sitecn/product_info.aspx?id=207350) |
|  | Mature miRNA sequences: AAGGGAGGAGGAGCGGAGGGGCCCU |  |
| hsa-miR-29a-3p | Bulge-Loop™hsa-miR-29a-3p qRT-PCR Primer Set, Standard, 100rxns | [miRQ0000086-1-1](http://www.ribobio.com/sitecn/product_info.aspx?id=200706) |
|  | Mature miRNA sequences: UAGCACCAUCUGAAAUCGGUUA |  |
| hsa-miR-29b-3p | [Bulge-Loop™hsa-miR-29b-3pqRT-PCR Primer Set, Standard, 100rxns](http://www.ribobio.com/sitecn/product_info.aspx?id=200734) | [miRQ0000100-1-1](http://www.ribobio.com/sitecn/product_info.aspx?id=200734) |
|  | Mature miRNA sequences: UAGCACCAUUUGAAAUCAGUGUU |  |
| hsa-miR-200c-3p | [Bulge-Loop™hsa-miR-200c-3pqRT-PCR Primer Set, Standard, 100rxns](http://www.ribobio.com/sitecn/product_info.aspx?id=201330) | [miRQ0000617-1-1](http://www.ribobio.com/sitecn/product_info.aspx?id=201330) |
|  | Mature miRNA sequences: UAAUACUGCCGGGUAAUGAUGGA |  |
| hsa-miR-4423-5p | [Bulge-Loop™hsa-miR-4423-5pqRT-PCR Primer Set, Standard, 100rxns](http://www.ribobio.com/sitecn/product_info.aspx?id=206926) | [miRQ0019232-1-1](http://www.ribobio.com/sitecn/product_info.aspx?id=206926) |
|  | Mature miRNA sequences: AGUUGCCUUUUUGUUCCCAUGC |  |
| hsa-miR-335-3p | [Bulge-Loop™hsa-miR-335-3pqRT-PCR Primer Set, Standard, 100rxns](http://www.ribobio.com/sitecn/product_info.aspx?id=203242) | [miRQ0004703-1-1](http://www.ribobio.com/sitecn/product_info.aspx?id=203242) |
|  | Mature miRNA sequences: UUUUUCAUUAUUGCUCCUGACC |  |
| hsa-miR-142-3p | Bulge-Loop™rno-miR-142-3p qRT-PCR Primer Set, Standard, 100rxns | miRQ0000848-1-1 |
|  | Mature miRNA sequences: UGUAGUGUUUCCUACUUUAUGGA |  |
| 5S | 5S rRNA qPCR Primer Set, Stardard | MQP-0302 |
|  | Human, mouse, rat universal 5S qPCR internal reference primers, product length of 91bp |  |

**Table 2. Screening of miRNAs Regulating CD133 Expression.**

| ID | Seq | Chr |
| --- | --- | --- |
| hsa-miR-29a-3p | UAGCACCAUCUGAAAUCGGUUA | ChrX |
| hsa-miR-29b-3p | UAGCACCAUUUGAAAUCAGUGUU | Chr3 |
| hsa-miR-200c-3p | UAAUACUGCCGGGUAAUGAUGGA | Chr16 |
| hsa-miR-4423-5p | AGUUGCCUUUUUGUUCCCAUGC | Chr1 |
| hsa-miR-335-3p | UUUUUCAUUAUUGCUCCUGACC | Chr14 |
| hsa-miR-142-3p | UGUAGUGUUUCCUACUUUAUGGA | chr5 |
| hsa-miR-142-5p | CAUAAAGUAGAAAGCACUACU | chr5 |
| hsa-miR-22-3p | AAGCUGCCAGUUGAAGAACUGU | chr15 |
| hsa-miR-30a-5p | UGUAAACAUCCUCGACUGGAAG | chr13 |
| hsa-miR-30e-5p | UGUAAACAUCCUUGACUGGAAG | chr6 |
| hsa-miR-377-3p | AUCACACAAAGGCAACUUUUGU | chr20 |
| hsa-miR-4739 | AAGGGAGGAGGAGCGGAGGGGCCCU | chr11 |
